# Interobserver Agreement between On-Call Radiology Resident and General Radiologist Interpretations of CT Pulmonary Angiograms and CT Venograms

**DOI:** 10.1371/journal.pone.0126116

**Published:** 2015-05-04

**Authors:** Bahar Tamjeedi, José Correa, Alexandre Semionov, Benoît Mesurolle

**Affiliations:** 1 Department of Radiology, McGill University Health Center, Montreal, Quebec, Canada; 2 Department of Mathematics and Statistics, McGill Statistical Consulting Service, Montreal, Quebec, Canada; Sapienza University of Rome, ITALY

## Abstract

**Objectives:**

To evaluate the interobserver agreement (IOA) between the initial radiology resident and the final staff radiologist reports of combined computed tomographic pulmonary angiograms (CTPA) and computed tomographic venograms (CTV) performed during on-call hours.

**Materials and Methods:**

Approval by the institutional review board was obtained. Six-hundred and ninety-six consecutive studies (CTPA or CTPA with CTV) performed during on-call hours and interpreted by 30 residents were identified. Radiology residents’ reports were compared to the final staff reports. Three tests outcomes were considered (positive, P; negative, N; indeterminate, I). Discordant cases were reviews by a chest radiologist.

**Results:**

CTPAs were reported by staff radiologists as positive for pulmonary embolism (PE) in 18% (126/694), with a kappa of 0.81 (95% CI 0.77-0.86) with 3 outcomes (P, N, I), and a kappa of 0.89 (95% CI 0.85-0.94) with 2 outcomes (P, N). Regarding PE location, good concordance was observed for positive studies, with a kappa of 0.86 (95% CI 0.78 – 0.95). CTVs were reported as positive by staff radiologists in 8.5% (33/388), with a kappa of 0.66 (95% CI 0.55-0.77) with 3 outcomes (P, N, I), and a kappa of 0.89 (95% CI 0.8-1.0) with 2 outcomes (P, N). The IOA between residents and staff radiologists increased with increasing residency year level for CTPAs, but did not for CTVs.

**Conclusions:**

Very good and good IOA were observed between resident and staff radiologist interpretations for CTPA and CTV, respectively, with tendency towards improved IOA as residency level of training increased for CTPA, but not for CTV.

## Introduction

Acute pulmonary embolism (PE) has long been recognized as a life-threatening emergency, and although the statistics vary widely depending on the clinical setting [1], associated mortality rates have been estimated at 100 000 deaths per year in the United States, including 15% of in-hospital deaths [2]. Consequently, the ability to obtain rapid and accurate diagnosis of PE becomes critical for clinical management, namely, early initiation of anticoagulant therapy.

Current clinical practice algorithms advocate use of computed tomographic pulmonary angiogram (CTPA) to reliably diagnose or exclude PE [1]. Most commonly, PE results from deep venous thrombosis (DVT). Therefore, in addition to the diagnosis of PE, the evaluation of the lower limbs in order to detect the presence of DVT is important for appropriate patient management. Although DVT can be diagnosed or excluded via sonographic evaluation of the lower limbs with Doppler technique, the use of combined CTPA and computed tomographic venogram (CTV) for one-time imaging allows diagnosing both PE and DVT [3]. PIOPED II study, combining CTPA and CTV showed increased sensitivity for PE from 83 to 90% and similar specificity (95%) [4, 5].

In situations where prompt diagnosis of PE is required, imaging studies are often performed during on-call hours, when radiology residents are the initial interpreters. Several studies have examined the agreement of resident reporting of on-call CTPA with morning staff radiologist reports, however these have either excluded CTV from their assessment or involved small numbers of patients, and none made use of interpretations by chest radiologist blinded to the preliminary and final reports as an additional reference [2, 3, 6–8].

We conducted a large retrospective study in order to (a) assess the interobserver agreement (IOA) between initial on-call resident reports and final staff radiologist reports of combined CTPA and CTV performed for diagnosis of thromboembolic disease during on-call hours, particularly according to residency level and (b) to evaluate IOA between staff and residents regarding PE location in cases of positive PE studies.

## Materials and Methods

This retrospective study was approved by “The Research Institute of the McGill University Health Centre”, which waived the need for informed consent.

### Patient Selection and Review of Radiology Reports

Patient population selection was performed by identifying through the picture archiving and communication system (IntelePACS, version 3.7.1; Intelerad Medical Systems, QC, Canada) all consecutive CTPA studies performed alone or together with CTV study during on call hours (during the week from Monday through Friday, residents give preliminary interpretations between the hours of 5 pm and 8 am, and on the weekends all day long) at a tertiary care hospital from January 1, 2008 to August 31, 2009. A total of 696 CTPA were identified, including 393 studies combining CTPA and CTV.

All 696 CTPA studies had a resident interpretation and 694 had a staff interpretation. Among the 393 CTV studies, 388 had a staff interpretation and 367 a resident interpretation with a total of 342 CTV interpreted by both a staff radiologist and a resident. Thus, the study population consisted of 694 CTPA and 342 CTV studies read by both residents and staff radiologists.

### Scan Technique and Interpretation

All studies were performed on GE Lightspeed 16-detector or 64-detector CT scanners (LightSpeed Pro, LightSpeed VCT, LightSpeed Discovery CT750 HD; GE Healthcare, Milwaukee, WI, USA). When CTPA was performed without CTV, patients received a bolus varying between 50–80 mL of non-ionic contrast (Iohexol [Omnipaque 300 mg/mL]; GE Healthcare Canada, Mississauga, ON, Canada) followed by a 35 mL saline flush through the antecubital veins at an average rate of 4.5 mL/s. The exact volume of contrast medium used was calculated by summing the time to peak and the scan time and multiplying the resulting time by the injection rate. When the studies were performed as combined PCTA and CTV, a bolus of 100 mL of non-ionic contrast (Iohexol [Omnipaque 300 mg/mL], followed by a 35 mL saline flush, were administered.

The scan delay was predetermined by a test bolus (20 mL) using the pulmonary trunk as the region of interest. CTPA scans were obtained using 1.25 mm collimation at 0.9 mm intervals, in a caudocranial direction from the diaphragm to the lung apices, during suspended full inspiration. Sagittal and coronal MIPs at 10x7 mm were automatically obtained in soft tissue (WW 400 WL 40) and lung (WW 1600 WL—600) windows. CTV was performed 180 seconds after administration of the IV contrast, from diaphragm to mid-calf in a craniocaudal direction, with the following scan parameters: 120 kVp; 180 mAs; detector width, 2.5 mm; rotation time, 1 second; table speed, 27.5 mm per rotation. Images were reconstructed at 5-mm section thickness without intersection gap.

According to our protocol, CTPA studies only were performed for patients under the age of 50 years. CTPA with a CTV were routinely performed for patients 50 years or older.

### Readers

A total of 30 different residents interpreted the studies: 7 residents in their second year of training (Postgraduate year PGY 2), 9 in their third year (PGY 3), 10 in their fourth year (PGY 4) and 4 in their fifth year (PGY 5). A total of 13 attending fellowship-trained staff radiologists, 5 from the thoracic section and 8 from the body imaging section, were involved in the study.

A fellowship-trained thoracic radiologist with 2 years’ experience as staff, who was blinded to the results, independently verified all discrepant CTPA and CTV studies, reported positive by the resident and negative by staff, and all discrepant CTPA and CTV studies reported negative by the resident and positive by staff. Indeterminate cases and the characteristics of the PE or DVT were not reviewed. He evaluated possible causative factors of the discrepancies, including patients factors (breathing and cardiac pulsations), technical factors (motion artifacts), and secondary pathologies (mass, consolidation, atelectasis and pleural effusion in the region of suspected PE). His impressions were recorded and analyzed for agreement with the initial resident and staff interpretations.

### Interpretation of Studies

For both CTPA and CTV the presence of a partial or complete endoluminal filling defects in the pulmonary arteries and deep venous structures of the abdomen, pelvis and legs, were considered diagnostic of pulmonary embolism or deep venous thrombosis, respectively [9, 10].

### Review of Radiology Reports

Interpretations of CTPA and CTV were classified as “positive”, “negative” or “indeterminate”, based on the preliminary residents’ reports and final radiologist staff reports in concurrent cases. The location of PE was recorded.

### Statistical Analysis

Patient records and information were anonymized and de-identified prior to analysis. The preliminary interpretation by the radiology resident on call was compared to the final report from the radiology staff the next day. In the first instance, three possible outcomes were considered: positive, negative, and indeterminate, and in the second, indeterminate cases were excluded from the residents’ reports section. In both instances, we evaluated inter-rater agreement via an unweighted Kappa coefficient, with 95% confidence interval (CI) [11]. As recommended by other researchers [12–14], along with kappa we also report observed proportions of agreement by category (positive, negative, indeterminate) and test for marginal homogeneity between the ratings from residents and staff radiologists with the Maxwell-Stuart Chi-square (χ^2^) test [15, 16]. This is done because the kappa coefficient alone is appropriate, if the marginal totals are relatively balanced (marginal homogeneity), but if the prevalence of a given response is very high or low, the value of kappa may paradoxically be low even when the observed proportion of agreement is quite high. The same procedure was employed to investigate the agreement of the results of the CTV examinations, used when the study was inconclusive. Additionally, a sensitivity analysis was done by stratifying the results by the year of residency of the first assessor. Kappa values were computed using SAS 9.2 (SAS Institute, Cary NC). The Maxwell-Stuart tests were done using the *R* software [17]. Statistical tests of hypothesis were two-sided and performed at the 0.05 level of significance.

## Results

Of the 696 patients, 275 (39.5%) were male and 412 (60.5%) were female. The mean age of the patients was 57.85 (standard deviation 0.7) years.

### CTPA studies

Of the 696 CTPA evaluated by residents 128 (18.4%) were positive, 486 (70%) negative and 82 (12%) indeterminate. On the other hand, staff radiologists reported on 694 CTPA, of which 126 (18%) were positive, 493 (71%) negative and 75 (11%) indeterminate. Table 1 shows the cross-classification of CTPA evaluation results for those patients with interpretations available from both residents and staff radiologists. Among the 694 CTPA read by both residents and staff radiologists, the overall agreement rate was 91.4% (634 of 694), whereas there were a total of 60 discrepant interpretations between residents and staff radiologists, corresponding to a discrepancy rate of 8.6%. The proportions of agreement on the positive, negative and indeterminate readings were 0.89, 0.95 and 0.75, respectively. The Maxwell-Stuart test showed evidence of marginal homogeneity between the raters (χ^2^ (2 *df*) = 1.47, *p* = 0.48). A good agreement between residents and staff radiologists was noted with a kappa of 0.81 (95% CI 0.77–0.86). When excluding indeterminate cases and considering only the 596 readings either positive or negative, 21 (3.5%) discrepancies ("frank" discrepancy) were identified. The rate of agreement was 96.5%. The proportions of positive and negative agreement were 91.4% and 97.8%, respectively. There was evidence of marginal homogeneity (χ^2^ (2 *df*) = 0.49, *p* = 0.51), with a kappa of 0.89 (95% CI 0.85–0.94).

**Table 1:** Staff radiologists and residents interpretations of CT Pulmonary Angiograms regarding the pretsence of a pulmonary embolism.

		Staff radiologist interpretations			
		Positive	Negative	Indeterminate	
**Resident interpretations**	Positive	112	12	3	127
	Negative	9	463	13	485
	Indeterminate	5	18	59	82
**Total**		126	493	75	694

Table 2 shows the cross-classification of locations (right, left or both sides) for the 112 PE readings that were interpreted as positive by both residents and staff radiologists. The overall agreement rate was 92.0%. The agreements for “right”, “left” and “bilateral” were 88.0%, 93.0% and 92.0%, respectively. There was evidence of marginal homogeneity (χ^2^ (2 *df*) = 1.0, *p* = 0.61), with a kappa of 0.86 (95% CI 0.78–0.95).

**Table 2:** PE location according to Staff Radiologists and Residents interpretations.

		Staff radiologist			
		Left	Right	Bilateral	
**Resident**	Left	11	0	1	12
	Right	0	40	4	44
	Bilateral	2	2	52	56
**Total**		13	42	57	112

Out of 21 “frankly” discrepant CTPA interpretations, the thoracic radiologist blinded to the reported results agreed with the staff interpretation in 13 out of 21 (62%) cases and with the resident interpretation in 8 out of 21 (38%) cases. Seven out of 21 (33%) discrepant interpretations were associated with motion artifacts.

### CTV studies

Residents reported on 367 CTV studies, of which 27 (7%) were positive, 299 (82%) negative and 41 (11%) indeterminate. Staff radiologists reported on 388 CTV studies, of which 33 (8%) were positive, 336 (87%) negative and 19 (5%) indeterminate. None of the patients who underwent a CTV study showed any thrombus in the inferior vena cava.

Table 3 shows the results of the 342 CTV read by both residents and staff radiologists.

**Table 3:** Staff radiologists and residents interpretations of CTV regarding the presence of a deep venous thrombosis.

		Staff radiologist interpretations			
		Positive	Negative	Indeterminate	
**Resident interpretations**	Positive	23	3	0	26
	Negative	2	270	4	276
	Indeterminate	4	21	15	40
**Total**		29	294	19	342

The overall agreement rate was 90% (308/342), whereas there were a total of 34 discrepant interpretations between staff radiologists and residents, corresponding to a discrepancy rate of 10%. The kappa value was 0.66 (95% CI 0.55–0.77). We noted that observed rates of agreement for positive, negative and indeterminate readings were 83.6%, 94.7% and 50.8%, respectively, and that the Maxwell-Stuart test showed evidence of marginal heterogeneity (χ^2^ (2 *df*) = 15.21, *p* < 0.001).

When excluding indeterminate cases and considering only readings interpreted as positive or negative by both residents and staff radiologists, there were 298 cases with 5 (1.7%) “frank” discrepancies. The overall rate of agreement was 98.3%, with rates of agreement for positive and negative readings of 90.2% and 99.1%, respectively. There was evidence of marginal homogeneity (χ^2^ (1 *df*) = 0.2, *p* = 0.65), with a kappa of 0.89 (95% CI 0.80–0.99).

For the 5 discrepant CTV cases the thoracic radiologist blinded to the reported results agreed with the staff interpretation in 3 cases and with the resident interpretation in 2 cases.

No patient showed a thrombus in the inferior vena cava.

### Residency level

Results are presented in Table 4. The rate of agreement between residents and staff radiologists increased with increasing residency year level for CTPA interpretations, with a higher kappa for PGY-4&5 residents (0.90; 95% CI 0.82–0.98) compared with PGY-2 residents (0.72; 95% CI 0.63–0.80). For CTV interpretations, the agreement between residents and staff radiologists was less consistent, with a lower kappa for PGY-4&5 residents (0.64; 95% CI 0.38–0.90) compared with PGY-3 residents (0.71; 95% CI 0.56–0.86).

**Table 4:** Agreement between resident and staff radiologists according to the year of residency for PE and DVT diagnosis for the 3 outcomes (Positive, Negative, Indeterminate).

Kappa for CTPA by year of residency training			
Year of residency	Kappa	95% CI	Numbers of CTPA interpretations
PGY-2	0.72	0.63–0.80	264
PGY-3	0.86	0.80–0.92	293
PGY-4&5	0.90	0.82–0.98	132
**Kappa for CTV by year of residency training**			
Year of residency	Kappa	95% CI	Numbers of CTV interpretations
PGY-2	0.61	0.45–0.77	111
PGY-3	0.71	0.56–0.86	157
PGY-4&5	0.64	0.38–0.90	72

## Discussion

The rate of positive PE results of CTPA in our study was higher than that reported in the literature—18% compared to 10% as, for example, reported by Raja et al [18]. We hypothesize that the higher rate of positive PE results observed might be due to inclusion of only on-call examinations in our study, as these cases probably have a higher positive pre-test probability. This is likely secondary to a perceived belief in limited imaging resources at our institution after regular office hours, which results in only the patients with a very high level of clinical suspicion of PE to undergo a CTPA study during on call hours. The rate of indeterminate CTPA studies of 10.8% at our institution is similar to 10.8% rate reported by Courtney et al [19] and 6% reported by the PIOPED II study [5].

The rates of CTV studies positive for DVT were 7.36% when interpreted by the resident and 8.51% when interpreted by the staff radiology in our study, compared to a lower positive rate of 5.5% reported by the PIOPED II study [4, 20]. Again, we hypothesize that the higher positive CTV rate in our study might be due to the fact that only on-call studies have been included and therefore the selected patients had a higher pre-test probability for DVT. The rates of indeterminate CTV in our study were 11.17% when interpreted by the residents, and 4.9% when interpreted by the staff radiologists, compared with 20.4% reported in the literature [20]. The significant difference in the rates of indeterminate CTV studies between residents and staff radiologists is interesting. Indeed, the rate of indeterminate CTV studies interpreted by the residents (11.17%) at our institution is closer to what is quoted in the literature (20.4%) than the rate of indeterminate CTV studies interpreted by the staff radiologists (4.9%). Our hypothesis is that staff radiologists at our institution might more readily dismiss indeterminate cases as negative due to the lack of long-term experience in the interpretation of CTV studies. Indeterminate interpretations have been associated with suboptimal contrast opacification of the deep veins, since with an adequate technique of opacification, the difference in attenuation between the clot and the blood should be obvious enough for a confident diagnosis [21, 22]. Arakawa et al. have shown that the amount of contrast, body weight and scanning delay were the 3 factors associated with adequate contrast opacification of the deep veins [22]. However, we did not investigate these parameters in our study. Finally, according to Cham, 17% and 6% of their CTVs were of fair or poor quality, respectively [21]. Although, we did not evaluate the quality of the CTVs in our study, we presume that the rate of indeterminate examinations is probably closely linked to the rate of technically fair or poor examinations.

We examined the concordance between the initial interpretations given by the radiology residents and the final interpretation made by the staff radiologist the next day, in patients suspected of having pulmonary embolism. We hypothesized that interobserver reliability would be higher for PE detection than for DVT detection, and that interobserver reliability will improve for both PE and DVT detection with increasing experience of the resident on call.

There have been several previous studies addressing the degree of agreement between experienced radiologists and radiology residents’ interpretations of CTPA in an emergency context [23–25]. Our study shows a good IOA between the on-call radiology residents’ and the staff radiologists’ interpretation for PE with an overall agreement of 91.4% (kappa of 0.81). This is concordant with study of Shaham et al. that found residents' preliminary interpretations of CTPA performed on call reasonably accurate (kappa statistics 0.7 and 0.8), indicating that preliminary interpretations by residents of PE studies are reasonably accurate [26]. Similarly, Gimberg et al. reported an overall agreement of 93% (kappa 0.8) between radiology fellows and radiology faculty in interpretations of CTPA [27]. Yavas et al., although reporting a good, but slightly lower correlation (kappa statistic of 0.7) between residents and experienced radiologists, suggests not basing the final long-term treatment only on the resident's reading [28]. Our results are also valid regarding PE locations since, if the study was interpreted as positive, there was good concordance with respect to the location of the pulmonary embolism between the staff and the resident on call.

Discrepancy rate in our study, which relied on imaging performed on 64-slice CT and 16-slice CT scanners, was 8.6% for CTPA interpretations, which is slightly lower than the 11.6% rate recently reported by Joshi et al, based on imaging performed on 64 slice-CT scanner [25]. Rufener noted less discordance using a 16-slice CT (6%) compared with a 4 slice-CT (20%) [29]. Yavas noted that discrepant cases were mostly due to motion artifacts [28]. Courtney noted that discrepancies occur for distal or minimally occlusive clots [19], similarly to Patel who identified a stepwise reduction in kappa values from segmental to subsegmental PE interpretations [30]. According to our review of discrepant cases (21 cases), 1/3 were related to respiratory motion artifacts, whereas 2/3 were not related to an identifiable cause. Although not investigated in our study, other technical causes, such as poor contrast enhancement and image noise could be also responsible for these discrepant cases [31].

With respect to residents’ experience, as the residency level increased, there was improved interobserver agreement between the resident’s and the staff radiologist’s interpretation of CTPA—senior residents showing better agreement with staff radiologists than junior residents (0.90 versus 0.72). As indicated by Joshi et al, improvement in resident’s performance might be related to a better anatomy understanding, assessment of quality of studies and improved interpretation skills in subtle or complex cases [25]. In addition, on call work is important for radiology residents in order to gain experience and confidence in image interpretation [32–34]. Recent implementation in our department of a double reading system for junior residents on call, supervised by a more senior fellow could potentially balance the relative lack of experience of junior residents (this system was not implemented during the time period of our study).

Regarding CTV interpretations, our study showed excellent IOA with a low discrepancy rate of 1.7% between the on-call radiology residents’ and the staff radiologists’ interpretations for DVT, if indeterminate studies were excluded. With inclusion of indeterminate studies (11% indeterminate studies), IOA decreased to 0.66 and discrepancy rate increased to 10%. Staff radiologists interpreting the CTV were body imaging and thoracic radiologists. These results are close to the 12% discrepancy rate (k:0.59) reported by Garg between 2 experienced thoracic and body imaging radiologists in the interpretation of CTV part of combined CTPA and CTV examinations, as was done in our study [35]. In the same vein, the IOA between residents and staff, broken down by year of training, does not reflect an improvement in agreement for the CTV results. This may reflect the added difficulty in CTV interpretation and raise the question of the proper imaging subspecialty assignment for CTV interpretation. Indeed, Branstetter has shown that subspecialist interpretations in emergency departments were the more accurate, but that subspecialists interpreting examinations outside their area of expertise had error rates similar to radiology residents [36].

Of 21 discrepant CTPA cases reviewed by the thoracic radiologist blinded to the preliminary and final reports, in 62% of cases the thoracic radiologist agreed with the staff and in 38% of cases the chest radiologist agreed with the resident. Of 5 discrepant CTV cases: in 3/5 (60%), the thoracic radiologist agreed with the staff and in 2/5 (40%), the thoracic radiologist agreed with the resident. Given the very low numbers of discrepant cases, the power is limited to garner additional statistically significant information.

In our studies that contained a CTV in addition to CTPA, we did not identify any single patient with a thrombus in the inferior vena cava. Therefore the validity of scanning the entire abdomen for a CTV at our institution should be re-evaluated. This is in keeping with Reichert el al. study suggesting that the additional value of CTV of the pelvis performed after CTPA is negligible [37].

There were limitations to our study. One such limitation is that our data did not quantify the technical quality of the CT examinations, as this certainly would affect study interpretation and has been previously identified as a reason for discrepant cases between residents and attending radiologists [25, 38]. Another limitation is that the analysis of the data did not include separate assessments based on the subspecialty of the staff radiologists which could be useful, since studies have shown than subspecialized radiologists interpreting emergency examinations outside their area of expertise have similar error rates than radiology residents [36]. The discrepancies of the reported locations of PE were not analyzed and although this would not have altered the treatment of pulmonary embolism, as the patient would have been treated regardless, it would help to define any discrepancies in knowledge of pulmonary anatomy [25]. We did not report deep venous thrombosis (DVT) locations and did not analyze their potential role in false negative or positive CTV studies. Indeed, anatomic variants (e.g. veins duplications) or specific locations (e.g. gluteal veins) are known causes of false negative CTV studies [39]. We did not review the isolated cases of positive CTV (without associated positive CTPA) and did not analyze the influence of a positive result of a CTPA on the interpretation of a CTV (and vice versa) [40]. We did not compare the IOA between 64-slice and 16-slice CT scanners. Indeed the difficulties in the interpretation of the CTV might be due to technical limitations of the 16-slice CTs. We did not analyze the possibility of a CTV result being influenced by the result of the corresponding CTPA study. Finally, we did not cross-reference the results of our CTVs with Doppler Ultrasound results performed for some index patients after CTPA with CTV studies, in order to verify false-positive and false-negative rates of DVT diagnosed on CTV. However, according to the PIOPED II study Doppler US and CTV are considered equivalent for the diagnosis of deep vein thrombosis and other factors such as radiation dose, cost and urgency of the need for a result are factors of consideration for which modality to use [4].

## Conclusion

Our study shows very good and good interobserver agreement between the on-call radiology residents’ interpretation and that of the staff radiologists for both PE and DVT, respectively. In addition, as the residency level increases, there is tendency towards improvement of the interobserver agreement between the resident and the staff for PE. However, the interobserver agreement between residents and staff broken down by year of training does not reflect an improvement in agreement for the CTV results, which may reflect the added difficulty in CTV interpretation.

## Supporting Information

S1 File Supporting data. This file contains data supporting results presented in Tables 1, 2, 3 and 4.
(XLSX)
